# The contribution of livestock to urban resilience: the case of Bamako, Mali

**DOI:** 10.1007/s11250-018-1651-2

**Published:** 2018-06-29

**Authors:** Lisa Crump, Stephanie Mauti, Abdallah Traoré, Alexandra Shaw, Jan Hattendorf, Jakob Zinsstag

**Affiliations:** 1 Swiss Tropical and Public Health Institute, Socinstrasse 57, P.O. Box, 4002 Basel, Switzerland; 2 University of Basel, Petersplatz 1, 4003 Basel, Switzerland; 3 Laboratoire Central Vétérinaire, Km 8, Route de Koulikoro, BP, 2295 Bamako, Mali; 4 Avia-GIS, Risschotlei 33,, 2980 Zoersel, Belgium

**Keywords:** Urban livestock, Livestock population, Resilience, Sheep, Poultry, Bamako

## Abstract

Urban livestock keeping is increasing in many sub-Saharan African cities, but detailed contextual information on its extent, challenges, and potential is limited. A cross-sectional household study was done in 2010 in Bamako, Mali. Thirty-two of 67 quarters were randomly selected with selection probability proportional to the size of the human population of the communes. Questionnaire interviews were done with a head of household in 1141 households, comprising 19,816 people in total. Sheep were kept by 16% (95% CI 14–18), while 21% (95% CI 17–24) kept poultry. The sheep to human ratio was 4:100, with an extrapolated city-wide population of 67,636 sheep (95% CI 61,018–75,595). The poultry to human ratio was 11:100, with an extrapolated city-wide population of 191,802 chickens (95% CI 176,212–208,772). For urban livestock holders, household-level enterprise gross margins were calculated for sheep production at USD 103 and poultry production at USD 50 annually. The annual gross margin was estimated at USD 35 per sheep and USD 17 per chicken. Based on these figures, the city-wide urban livestock total gross margin for Bamako in 2010 was estimated at USD 5.6 million. Detailed population data help clarify the urban livestock animal human interface in diverse contexts and highlight the important contributions that urban small-holder production adds to food security and resilience. The potential for urban livestock production informs decision-makers in developing adapted, sustainable policies in resource-constrained environments.

## Introduction

According to the World Urbanization Prospects report (United Nations 2004), Africa was the least urbanized region at the midpoint of the last century, with 15% of total population living in urban areas. However, since the 1970s, sub-Saharan Africa had very high growth in the urban population share, reaching 40% by 2014. By 2050, it is projected that more people (56%) will live in urban than rural areas in Africa. According to the latest Malian national census in 2009 (R.G.P.H. 2009), 22% of the total population lived in urban settings and more than 55% of this urban population resided in the district of Bamako. UNICEF data for 2012 (https://www.unicef.org/infobycountry/mali_statistics.html, accessed on March 23, 2017) reported the urban population share in Mali at 36%. The average annual growth rate of the urban population was estimated at 4.7% for the previous 20 years and forecast to continue at 4.8% until 2030. The gross national income per capita in 2012 was 730 USD, with more than 50% of the population living below the international poverty line of 1.25 USD per day from 2007 to 2011 (World Bank, http://data.worldbank.org/country/mali, accessed on February 16, 2018). Although there are different definitions, drivers, and features of urbanization (Hove et al. 2013), indisputably such rapid growth and shifts have important economic, social, and physical repercussions which test the well-being and resilience of populations.

Agriculture provides approximately 40% of the gross domestic product (GDP) in Mali (IndexMundi 2014), with the combined value of livestock products contributing about half of agricultural GDP (FAO 2014). Livestock production systems are highly diverse, ranging from transhumant to sedentary and rural to urban throughout the country (Amanor 1995). Livestock contribute to alleviate poverty and reduce vulnerability in rural communities and play multiple, complex roles as social and biological capital (Alary et al. 2011).

Urban and peri-urban agriculture (UPA), which has been defined as crop cultivation and animal husbandry for food within or around cities (Mougeot 2000), is increasingly practiced throughout sub-Saharan Africa as a consequence of this rapid urbanization (Drechsel and Dongus 2010). Numerous studies describe the social roles, economic functions, environmental benefits, and associated problems (de Bon et al. 2010; Graefe et al. 2008; McMichael 2000), but the West African context remains markedly diverse. To facilitate comparisons between cities and make recommendations for innovative technologies and policies, the UPA production systems were classified to identify a regional typology (Dossa et al. 2011a) which included subsistence and commercial gardening and livestock production. Livestock keeping was shown to be the most popular activity among UPA households in West Africa, with 62% of households involved in animal husbandry (Amadou et al. 2012). Although urban livestock keeping has been considered to be an indication of poverty (Schiere and van der Hoek 2001), more recent work in Ouagadougou, Burkina Faso, found that in the 26% of households which kept livestock, diverse reasons were stated, including a survival strategy for poor urban dwellers and also increased income by established city residents (Thys et al. 2005). Accurate information on household income and expenditures is difficult to obtain in sub-Saharan Africa (Benin and Randriamamonjy 2008), so proxies for socioeconomic status were assessed in cities in Burkina Faso, Nigeria, and Mali with the conclusion that urban agriculture is a livelihood strategy practiced across all socioeconomic groups (Dossa et al. 2011b). However, the primary motivations vary largely across socioeconomic groups. Poor households seek to improve cash income flow and nutritional status (Cissé et al. 2005) while middle and higher income families aim to save on food expenditures and diversify income sources (Binns and Lynch 1998; McClintock 2010).

Despite increased practice and prominence in West Africa, detailed information on the extent of urban livestock production and its challenges and potentials is not available for many areas (Nasinyama et al. 2006). This paper reports the results of a cross-sectional household study assessing the urban livestock and companion animal population across Bamako, Mali. The potential economic contribution of urban livestock production to household income is estimated.

## Material and methods

### Study site

This study was embedded in a larger cross-sectional study in Bamako, the capital of Mali, located in the southern part of the country. The city is divided in 6 communes with a total of 67 quarters. The national census recorded 1.8 million inhabitants in Bamako (RGPH 2009). The city has a sub-humid savannah climate with an average temperature above 30 °C each month. The total surface area of the district of Bamako is about 267 km^2^ (UN-HABITAT 2010).

### Sampling

Questionnaire interviews were conducted in all six communes of Bamako between May and June 2010. A household register was not available, so we used a multi-stage cluster technique to obtain a representative sample (Schelling and Hattendorf 2015). We randomly selected 32 out of a total of 67 quarters with selection probability proportional to the size (PPS) of the human population of the communes. Commune population was used because reliable data on the quarter level was not available. The basic sampling unit was set as a compound, which was defined as all of the houses surrounded by a wall and could include more than one household.

Five field teams, each consisting of one veterinary officer and one interviewer, started in each selected quarter at the compound of the quarter chief, spun a bottle, and walked 200 m in the indicated direction, where they flipped a coin to select one side of the road for the inclusion of two neighboring compounds. This procedure was repeated until at least 38 compounds per quarter were enrolled. In each sampled compound, information on one single household was obtained, whenever possible from the head of household.

### Data collection

Prior to the study, meetings were held with the local chiefs of each quarter. The questionnaire was tested in a non-selected quarter. The location of each compound was recorded using a global positioning system (GPS). The questionnaire included questions about number of household members, occupation of the head of household and religion, and ethnicity of the interviewee. The building construction of the wall and roof of the house was observed by the field team during the interview. In addition, questions about total numbers and ownership of livestock and companion animals were asked. When possible, interviewers visually noted animals present in the household, although in some cases, animals were elsewhere. The questionnaire was in French, and questions were translated into Bambara when necessary. Informed consent was obtained prior to each interview.

### Data entry and statistical analysis

Data was double-entered in Microsoft® Access 2010 and checked for inconsistencies using Epi Info™. Statistical analyses were done with STATA IC 12. Questionnaire data was analyzed with generalized estimating equation models, taking clustering into account. The extrapolation of the total livestock population of Bamako was done using a negative binomial model to predict the point and interval estimate of the mean livestock per capita. These numbers were then multiplied by the total human population of Bamako, as counted during the nationwide census in 2009 (RGPH 2009).

### Economic contribution

We calculated gross margins for small-holder urban livestock keeping enterprises to estimate the contribution of livestock to household income, as described by Rushton (2008). As sheep and poultry were the main species recorded in Bamako (Table 1), small-scale urban livestock holders in this context were assumed to be engaged in two substantive enterprises: production of broiler chickens and production of whole carcass sheep, including animals produced for Tabaski religious and holiday celebrations. The gross margin was selected as a good indicator of income for this type of activity where there are few fixed cost overheads. It is calculated individually for each farm enterprise and comprises output less variable costs. For livestock, the output valuation must include not just sales and home consumption but also adjust for livestock purchases, gifts, transfers, and any change in the value of the flock/herd. Variable costs for sheep included shared costs for herder salary and vaccination and deworming costs*.* Nutritional needs are met with available grazing, so no purchased feed costs were included. The variable cost for poultry included only vaccination costs, since chickens are fed from kitchen waste. The gross margins for each enterprise were summed to obtain the total livestock gross margin. To calculate output, we considered goods produced (sheep/broilers sold plus home consumption of livestock; eggs are not consumed nor sold). Herd/flock sizes and value were assumed to remain stable with average livestock holdings remaining much the same, as older breeding animals were culled and replaced by their offspring. Gross margins were calculated for a typical household herd/flock size and then the average gross margin per household was worked out and from this extrapolated to the relevant urban livestock population.

**Table 1:** Urban animal keeping (*n* = 1141)

		Number of households	Percentage of animal-households (95% CI)	Median animal/animal-keeping hh (IQR)
Livestock				
	Sheep	180	15.7% (13.5–18.3)	3 (1–5)
	Poultry	235	20.5% (17.2–24.3)	8 (4–12)
Pets				
	Cats	52	4.5% (3.4–6.1)	1 (1–2)
	Dogs	125	10.9% (8.7–13.6)	1 (1–1)

### Sheep output

According to Malian national statistics, 75% of sheep in an average herd are adult females with a fertility rate of 90% (Direction Nationale des Productions et des Industries Animales 2015) and a productive life of 4 years. One breeding ram is shared among the local community. On the local market in Bamako, the average value for a sheep is USD 55, which covers males and females and seasonal price variability associated with the religious holiday calendar (Direction Nationale des Productions et des Industries Animales 2015). Sheep are generally dewormed and vaccinated against pasteurellosis and peste des petits ruminants (PPR) (Amadou et al. 2012). In the local husbandry system, sheep graze available pasture or occasionally crop residues, with no additional concentrate typically provided. In urban areas, one herder manages a co-mingled group of about 50 sheep, with each family contributing 5 USD annually to the herder’s salary.

### Poultry output

In the local traditional system, eggs are not marketed or consumed, so all contribute to broiler production. One rooster is assumed per flock. It is assumed that hatchlings are equally distributed between male and female and they are sold for the same price. Broilers are marketed at 4 months of age for 5 USD on the local markets (Direction Nationale des Productions et des Industries Animales 2015). Females reach reproductive maturity at 6 months of age and are considered productive until 2 years of age. We assume an average clutch of four hatchlings every 2 months per producing hen. Survival to maturity is estimated at 18%. Fifty percent of surveyed flocks in Malian villages are vaccinated against Newcastle disease (Molia et al. 2015), so this was included as variable cost. Other costs were negligible as flocks are fed on household refuse without additional purchased feed.

## Results

### Livestock population

Interviews were conducted in 1141 households, comprising 19,816 persons in total. The mean number of people per household was 11. Overall, 40% (95% CI 37–44) of all households kept animals. Sheep were the most commonly held animal in Bamako, kept by 16% (95% CI 14–18), of the surveyed households with a median number of three animals per household. About 21% (95% CI 17–24) of the surveyed households kept poultry, with a median flock size of 8. Animals commonly kept in the surveyed households are listed in Table 1.

**Fig. 1:**
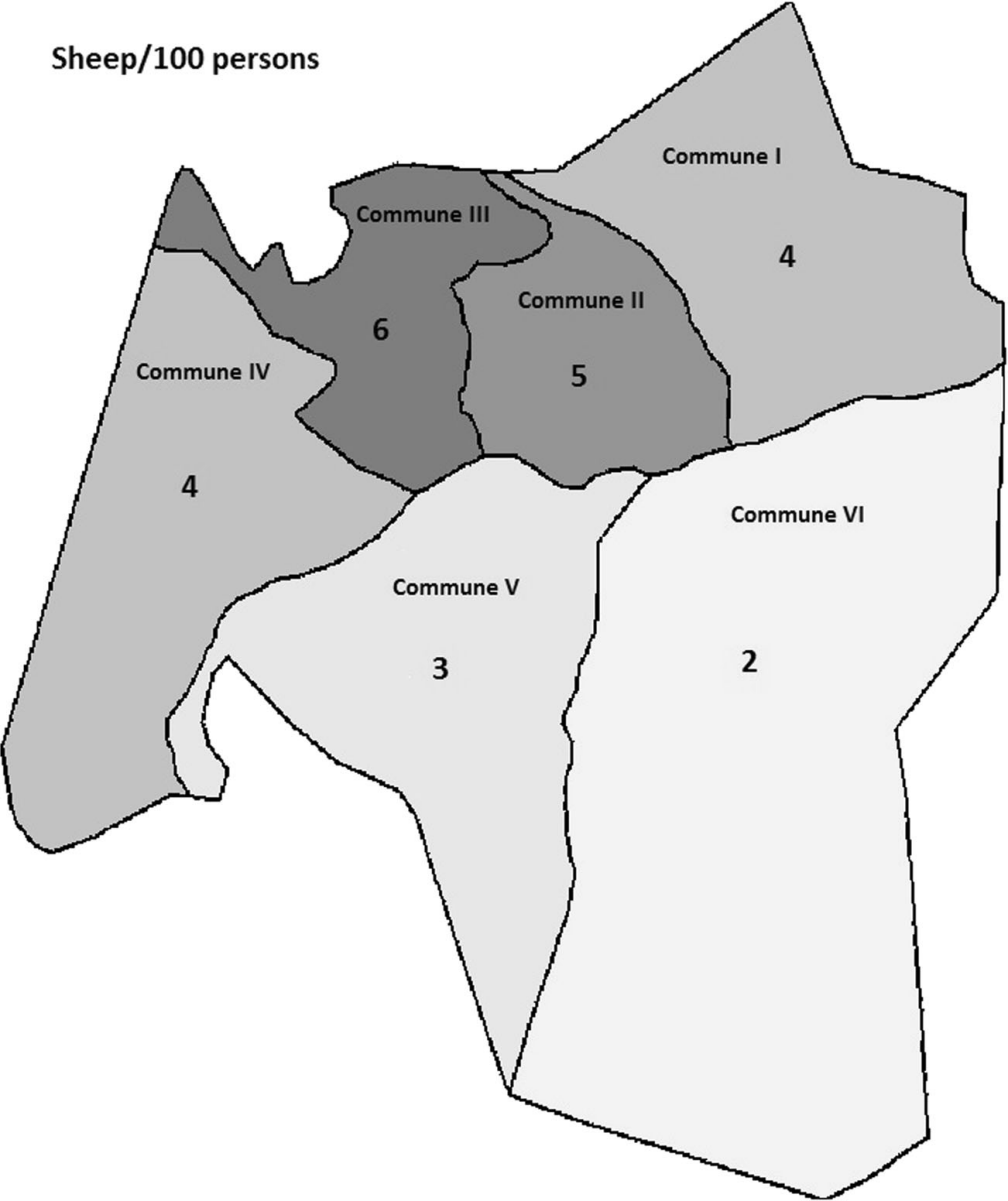
Sheep per 100 persons, by commune in Bamako, Mali

The sheep to human ratio was 4:100, which translates to an extrapolated population of 67,636 sheep (95% CI 61,018–75,595) for the entire city of Bamako (Table 2). The ratio of sheep to humans varied, ranging from 2 to 3 in the southern communes V and VI and from 4 to 6 in the northern communes I, II, III, and IV, as shown in Fig. 1.

**Table 2:** Extrapolation of the sheep population in Bamako

	*N* compounds	*N*	Mean	[95% Conf. interval]	
Sheep	1139	738	0.65	0.53	0.77
Humans	1139	19,816	17.40	16.59	18.21
Sheep per 100 persons^a^			3.72	3.46	4.00
Extrapolation based on the total human population (1,810,366 inhabitants) estimated in 2009					
Sheep			67,636	61,018	75,595

^a^Negative binomial model adjusted for within quarter correlation

**Fig. 2:**
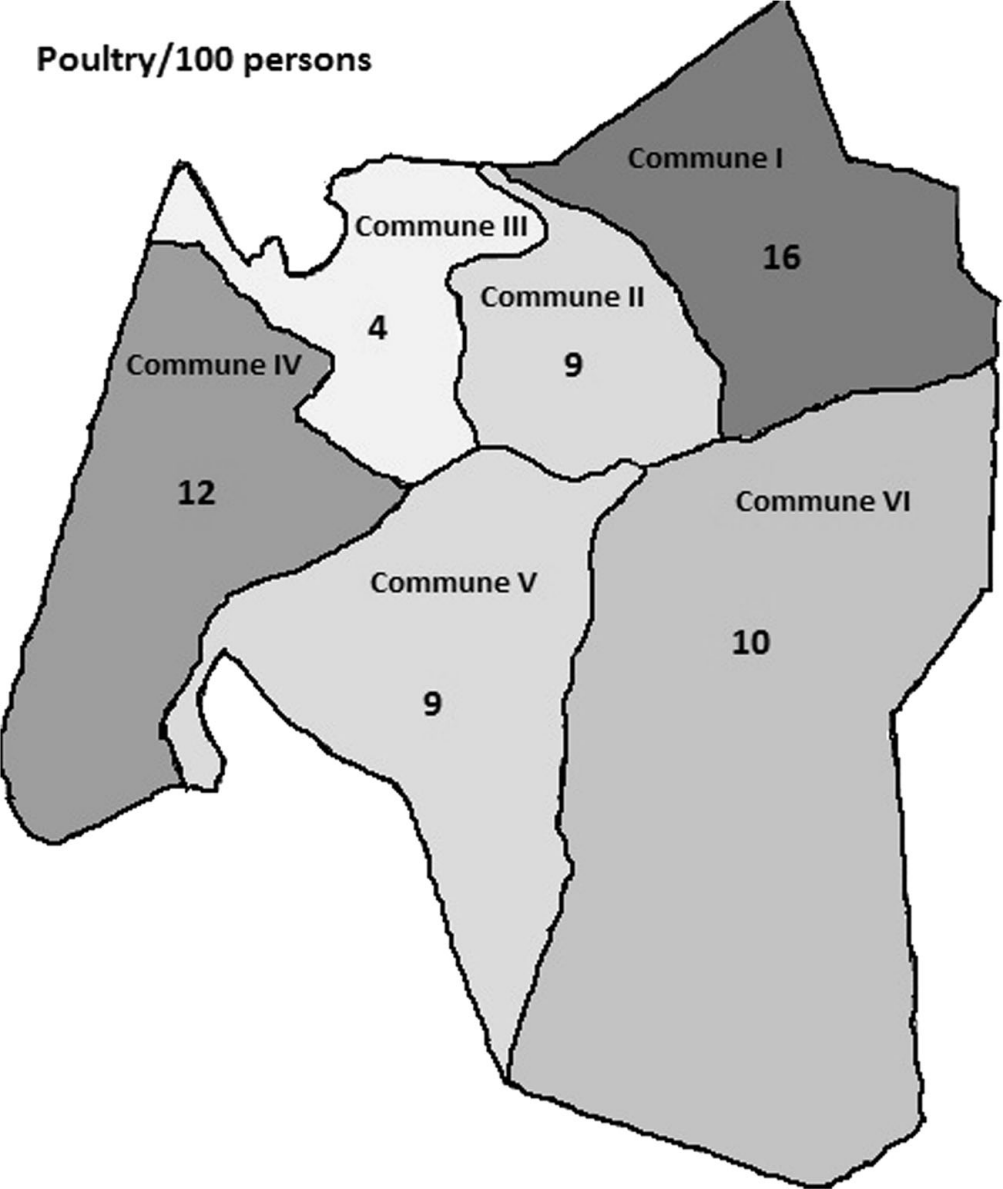
Poultry per 100 persons, by commune in Bamako, Mali

The poultry:human ratio was 11:100, while the extrapolated population of chickens was 191,802 (95% CI 176,212–208,772) across the city of Bamako (Table 3). Figure 2 depicts the ratio of poultry to humans across communes, which was high with the exception of commune III.

**Table 3:** Extrapolation of the poultry population in Bamako

	*N* compounds	*N*	Mean	[95% Conf. interval]	
Poultry	1141	2098	1.84	1.69	2.01
Humans	1139	19,816	17.40	16.59	18.21
Poultry per 100 persons^a^			10.59	9.73	11.53
Extrapolation based on the total human population (1,810,366 inhabitants) estimated in 2009					
Poultry			191,802	176,212	208,772

^a^Negative binomial model adjusted for within quarter correlation

### Predictors for livestock ownership

Predictors for sheep ownership are listed in Table 4. In bivariate analysis, only slight variations in sheep ownership among ethnic or religious groups were noted. Among the variables used as proxies for socioeconomic status, families with brick house walls more often kept sheep compared to families living in houses with cement walls (17 vs 6%, OR 0.3; 95% CI 0.1–0.9). The type of roof construction was not a significant predictor among sheep owners.

**Table 4:** Predictors for sheep ownership

Sheep ownership				
Predictor		Percentage of sheep-households (*n*/*N*)	*p*	OR (95% CI)
Occupation (*n* = 1119)	Private sector	15% (96/637)		Ref
	Public sector	17% (44/264)	0.575	1.1 (0.8–1.7)
	Agriculture	19% (10/52)	0.361	1.4 (0.7–2.8)
	Others	16% (26/166)	0.942	1.0 (0.6–1.6)
Religion (*n* = 1139)	Muslim	16% (175/1105)		Ref
	Christian	13% (4/30)	0.699	0.8 (0.3–2.4)
	Others	0% (0/4)	n.d.	n.d.
Ethnic group (*n* = 1130)	Bambara	17% (64/373)		Ref
	Malinke	13% (22/176)	0.167	0.7 (0.4–1.2)
	Peulh	14% (26/183)	0.374	0.8 (0.5–1.3)
	Sarakole/Soninke/Marka	22% (26/117)	0.277	1.3 (0.8–2.2)
	Sonraï	9% (4/43)	0.183	0.5 (0.2–1.4)
	Dogon	16% (7/44)	0.807	0.9 (0.4–2.1)
	Sénoufo/Minianka	16% (13/82)	0.722	0.9 (0.5–1.7)
	Bobo	17% (2/12)	0.935	0.9 (0.2–4.4)
	Others	15% (15/100)	0.559	0.8 (0.5–1.5)
Building construction (wall) (*n* = 1139)	Cement	17% (139/805)		Ref
	Mudbrick	6% (4/64)	*0.03*	*0.3* (*0.1–0.9*)
	Improved mudbrick	14% (37/270)	0.158	0.7 (0.5–1.1)
Building construction (roof) (*n* = 1137)	Steel sheet	16% (129/820)		Ref
	Straw	33% (1/3)	n.d.	n.d.
	Cement	16% (50/314)	0.916	1.0 (0.7–1.5)

*p* <0.05 is considered significant

Table 5 lists the predictors for poultry ownership. People living in houses with steel sheet roof were less likely to own poultry compared to people living in houses with cement roof (15 vs 23%, OR 0.6; 95% CI 0.4–0.8). Occupation, religion, ethnic group, and type of building wall were not significantly associated with poultry ownership.

**Table 5:** Predictors for poultry ownership

Poultry ownership				
Predictor		Percentage of poultry-households (*n*/*N*)	*p*	OR (95% CI)
Occupation (*n* = 1119)	Private sector	22% (137/637)		Ref
	Public sector	19% (50/264)	0.769	0.9 (0.7–1.4)
	Agriculture	25% (13/52)	0.464	1.3 (0.7–2.4)
	Others	18% (30/166)	0.332	0.8 (0.5–1.3)
Religion (*n* = 1139)	Muslim	20% (226/1105)		Ref
	Christian	27% (8/30)	0.559	1.3 (0.6–3.0)
	Others	0% (0/4)	n.d.	n.d.
Ethnic group (*n* = 1130)	Bambara	22% (83/373)		Ref
	Malinke	23% (40/176)	0.961	1.0 (0.6–1.5)
	Peulh	21% (38/183)	0.699	0.9 (0.6–1.4)
	Sarakole/Soninke/Marka	18% (21/117)	0.169	0.7 (0.4_1.2)
	Sonraï	19% (8/43)	0.494	0.8 (0.3–1.7)
	Dogon	25% (11/44)	0.799	1.1 (0.5–2.3)
	Sénoufo/Minianka	20% (16/82)	0.615	0.9 (0.5–1.5)
	Bobo	25% (3/12)	0.72	0.8 (0.2–3.3)
	Others	14% (14/100)	0.06	0.6 (0.3–1.0)
Building construction (wall) (n = 1139)	Cement	20% (159/805)		Ref
	Mudbrick	23% (15/64)	0.849	0.9 (0.5–1.8)
	Improved mudbrick	23% (61/270)	0.656	1.1 (0.8–1.5)
Building construction (roof) (n = 1137)	Steel sheet	23% (189/820)		Ref
	Straw	0% (0/3)	n.d.	n.d.
	Cement	15% (46/314)	*0.004*	*0.6* (*0.4–0.8*)

*p* <0.05 is considered significant

### Economic calculations

Using the median livestock numbers for commonly held animals in the surveyed households, we calculated the gross margins for production contributed to the economy in Bamako. At the household level, the annual livestock gross margin, specifically output less variable costs for sheep and poultry as detailed in Table 6a, was calculated at USD 153 per animal-owning household. Replacement of breeding stock was considered, according to national data (Direction Nationale des Productions et des Industries Animales 2015).

**Table 6:** Household livestock contribution to the Bamako economy

(a) Annual livestock gross margin per household						
	Sheep			Poultry		
Output	USD	110.00	3 sheep × 0.90 fertility rate × 0.75 producing females/herd^y^ ≥ 2 sheep produced × USD 55 market value	USD	50.00	3 hens × 4 hatchlings/hen × 0.85 producing hens/flocky ≥ 10 broilers × USD 5 market value
Variable cost	USD	− 6.80	(USD 0.60 vaccination − deworming cost/animal × 3 sheep) + USD 5 herder salary/household	USD	− 0.40	USD 0.05 vaccination cost/bird × 8 birds^z^
Gross margin	USD	103.20		USD	49.60	
(b) Annual livestock gross margin per animal						
	Sheep			Poultry		
Output	USD	37.13	0.90 fertility rate × 0.75 producing females/herd^y^ × USD 55 market value	USD	17.00	4 hatchlings × 0.85 producing hens/flock^y^ ≥ 3.4 broilers × USD 5 market value
Variable cost	USD	− 2.27	USD 0.60 vaccination − deworming cost/animal + USD 1.67 herder salary/animal	USD	− 0.04	USD 0.04 vaccination cost^z^
Gross margin	USD	34.86		USD	16.96	
(c) City-wide annual livestock gross margin						
	Sheep			Poultry		
Gross margin	USD	2,357,790.96	gross margin per animal (34.86) × extrapolated number of sheep (67,636)	USD	3,252,961.92	gross margin per animal (16.96) × extrapolated number of poultry (191,802)

Annual urban livestock gross margin: USD 153 per animal-owning household

Total calculated gross margin from sheep and chicken production city-wide per annum USD 5,610,753

^y^To account for replacement breeding stock

^z^Breeding stock only

To estimate the total contribution of urban livestock in Bamako, we calculated per animal gross margins for sheep USD 35 and poultry USD 17, as shown in Table 6b. Using the extrapolated animal population numbers, generated as above, we calculated city-wide gross margins, summing them to project the annual contribution from livestock-owning households to the city-wide economy, as seen in Table 6c. This yielded an estimate of USD 5.6 million for the annual city-wide economic contribution of urban livestock.

## Discussion

This study in Bamako was embedded in the EU-FP7 funded project Integrated Control of Neglected Zoonoses (ICONZ). Results of the research in Mali have been previously published (Mauti et al. 2015, 2016, 2017). In this study, we interviewed and collected data from only one household in each selected compound. Out of 1141 households surveyed in all six communes of Bamako, Mali, the most commonly kept livestock were sheep (16%) and poultry (21%). Although dogs were well represented in this study, they were mainly kept for protection (Mauti et al. 2017; Mindekem et al. 2005). Some ethnic groups (e.g., Bobo, Malinke) also consume dog meat (Mauti et al. 2017). Numerous West African studies have reported data on the livestock keeping aspect of urban and peri-urban agriculture, but the specific context seems to be a key determinant due to important differences in city size, structure, degree of urbanization, development history, and socioeconomic and cultural factors (Cissé et al. 2005). This was illustrated in our study, where there was variation in the number of animals held per 100 people (sheep ranging from 2 to 6, poultry ranging from 4 to 16) across the different communes. Undoubtedly, these numbers are influenced by natural, physical, and human-designed systems in the urban and peri-urban areas. It was interesting that commune III had the highest sheep:human ratio (16:100) and the lowest poultry:human ratio (3:100). We expected that people who had the resources to raise sheep would also engage with chicken farming. In Sikasso, the second largest city in Mali, about 60% of households were involved in animal husbandry, with two thirds keeping cattle and small ruminants and half keeping donkeys. Chickens were kept by four out of five households, with an average flock size of 18 (Amadou et al. 2012). In Ouagadougou, the capital and largest city of Burkina Faso, 26% of all households, which were broadly distributed throughout the city, were involved in livestock production, with about two thirds keeping poultry and one fifth keeping small ruminants (Thys et al. 2005). In Maradi, the third largest city in Niger, about 60% of urban households kept sheep or goats (Ali et al. 2003). Our results are most similar to those in Ouagadougou, in particular, that the distribution of animal keeping households was broad throughout the city, but there was overall a smaller percentage (one fifth versus two thirds) keeping poultry in Bamako. Although the size of households which engage in urban agriculture tends to be larger than those not producing, it is unclear whether urban farming allows support of larger households or whether poorer households are compelled to produce for food security (Prain and Lee-Smith 2010).

In this study, predictors for livestock ownership were not especially informative. Sheep owners were more likely to have cement house walls than mud brick, which could indicate higher incomes and more purchasing power. Poultry holders were less likely to have cement roofing, which could indicate less income. It could be that the cost barrier for owning animals other than chickens is too high for these families. But there could also be other important drivers, such as more access to open space in certain quarters, which might allow for sheep ownership, while more densely populated areas might only be suitable for poultry holding, or commercial areas with higher traffic might preclude husbandry of larger animals. Further work is needed to elucidate the contributing reasons.

In Mali, the National Directorate for Animal Production and Industries (NDAPI) was established in 2005 and compiles annual livestock production statistics from abattoir records. However, output from urban livestock is primarily sold on local markets and home slaughter is common because policy governing animal regulations is often poorly regulated or non-existent. Consequently, the national statistics are unlikely to establish accurate numbers for animals held in urban and peri-urban areas, instead reflecting the intensive sector large-scale producers and illustrating market shares.

Although a detailed economic evaluation of the contribution of small-holder livestock production to the livelihood of Bamako residents is beyond the scope of this study, we used data from the household survey, along with literature values and national data, to estimate a livestock gross margin calculation. Where it was not possible to find published values or stratified national data, we used values based on local knowledge. We assumed average local market prices; however, there is possibly some inflation in sale prices for the time periods surrounding Tabaski celebrations. According to species-stratified national data on monthly prices for animals sold at the main controlled Bamako markets in 2014, the average price for an adult male sheep was USD 143 with prices relatively stable throughout the year (Direction Nationale des Productions et des Industries Animales 2015).We used a lower average price of USD 55 per sheep sold, based on our knowledge of local markets, assuming household producers would be most likely to sell their animals at the nearest market. An average value for net output per ewe per year of USD 60, based on traditional farming systems in southwest Nigeria, a similar humid West African zone, was previously described (Upton 1985). Our calculation for the contribution of sheep in Bamako was less than that at USD 103 per household, or USD 34 per ewe owned. The published figure of USD 60 for net output per ewe was estimated more than 30 years ago and for our calculations, we have not adjusted with an inflation multiplier. Thus, we propose that our gross margin calculation, while estimated, may be lower than the actual value.

In Malian household flocks, 36% of birds are reported as older than 6 months (Molia et al. 2015), which is the age of reproductive maturity. Although our study did not collect data on flock age structure, applying this value to our average flock of eight birds would provide a similar estimate of productivity. For example, in an average flock of 8 birds, 0.36 producers × 8 birds, would yield 3 producing chickens. It follows that each flock retains one rooster, leaving four young birds which are fattening for market. The young birds are sold at 4 months of age; thus, a farmer would expect to produce three crops of four birds, with ten sold annually and two being retained as breeding stock replacement. This is confirmed by our calculation of 12 broilers annually produced per farming household. We used a value of USD 5 per broiler sold on the local market based on our local knowledge. National data from 2014 indicated an average price of USD 6 per cock sold at the main Bamako controlled markets, with noted variability in the monthly averages (Direction Nationale des Productions et des Industries Animales 2015). Therefore, our estimate is likely to be conservative.

There may be other costs/risks involved in urban livestock production that we have not considered in our estimate (e.g., “informal” permits required to access grazing grounds or animal loss from disease, being killed or theft). Despite these limitations, according to our calculation, sheep and poultry both contribute meaningfully to overall increase annual household income in animal-owning households. In the case of sheep (USD 103 annually), the increase is close to twice the amount contributed by poultry (USD 50 annually), but the importance of both sectors for Malian urban livestock producers is validated by these data.

Using aggregate data from 2006 to 2012, reported median annual household income in Mali was USD 1983, while median per capita income was USD 165 (Gallup 2013). According to our estimate for urban small holders, the total livestock gross margin is calculated as USD 153, potentially adding nearly 8% of median household income in Mali. Extrapolated onto a city-wide scale, the gross margin calculation for small-holder urban livestock production in Bamako for 2010 is USD 5.6 million. In the same time frame of 2007–2011, it was estimated that 50% of the Malian population lived below the international poverty line of USD 1.25 per day (UNICEF 2017). For urban livestock holders, the added household income, as we estimated for 2010, represents about one third of that amount, at USD 0.42 per day, representing a substantial increase. Because urban agriculture, including livestock production, constitutes a livelihood strategy practiced across all socioeconomic groups in cities (Dossa et al. 2011b) and has the potential to contribute meaningfully to household income, it should be considered as a resilience strategy. Continuing, or even increasing, civil unrest and political conflict in sub-Saharan Africa impacts livelihoods in myriad ways. Food security is affected, both locally and generally due to insecurity of trade routes. Expansion of the urban livestock production sector could provide valuable supplement for families in many locales.

Nonetheless, agriculture, including production of livestock, in cities is an inherently complex issue. There are constraints with use of and competition for limited resources, as well as implications for and necessary regulations on public health, animal health, and governance. Currently, poultry keeping is permitted, but local regulations prohibit keeping of cattle, sheep and goats within household compounds. However, livestock owners often do not comply, so dialog with community decision-makers might improve prevailing practices. Institutional change and policy recognition of the role and importance of agriculture will be increasingly well served through effective use of gender-sensitive stakeholder platforms. Urban and agricultural issues should be considered as integrated, and although data are scarce, recent work suggests that multidisciplinary approaches can contribute new understanding and significant results. A recent study on land use policy analyzed arrangements between actors, concluding that it was not political instruments that most affected urban agriculture but rather how they were interpreted and negotiated (Robineau 2015). In addition to community-level concerns, urban livestock producers also face constraints at the household level related to feed and forage access, healthcare knowledge/affordability, and safe waste management (Amadou et al. 2012; Thys et al. 2005).

## Conclusion

In Bamako, Mali, sheep and poultry were most commonly raised by urban livestock holders, with 16% (95% CI 14–18) and 21% (95% CI 17–24), respectively. Detailed population data clarify the urban livestock animal human interface in diverse contexts and may be used, along with economic estimates, to inform decision-makers in developing adapted, sustainable policies in resource-limited environments. Participatory stakeholder processes, involving communities and local authorities together with technical experts, have been shown to be effective in finding locally adapted, sustainable solutions, through urban animal agriculture, for food security, disease risks, and livelihood resilience.
